# The European Nucleotide Archive in 2024

**DOI:** 10.1093/nar/gkae975

**Published:** 2024-11-18

**Authors:** Colman O’Cathail, Alisha Ahamed, Josephine Burgin, Carla Cummins, Rajkumar Devaraj, Khadim Gueye, Dipayan Gupta, Vikas Gupta, Muhammad Haseeb, Maira Ihsan, Eugene Ivanov, Suran Jayathilaka, Vishnukumar Kadhirvelu, Manish Kumar, Ankur Lathi, Rasko Leinonen, Jasmine McKinnon, Lili Meszaros, Joana Pauperio, Stephane Pesant, Nadim Rahman, Gabriele Rinck, Sandeep Selvakumar, Swati Suman, Yanisa Sunthornyotin, Marianna Ventouratou, Zahra Waheed, Peter Woollard, David Yuan, Ahmad Zyoud, Tony Burdett, Guy Cochrane

**Affiliations:** European Molecular Biology Laboratory, European Bioinformatics Institute (EMBL-EBI), Wellcome Genome Campus, Hinxton, Cambridgeshire CB10 1SD, UK; European Molecular Biology Laboratory, European Bioinformatics Institute (EMBL-EBI), Wellcome Genome Campus, Hinxton, Cambridgeshire CB10 1SD, UK; European Molecular Biology Laboratory, European Bioinformatics Institute (EMBL-EBI), Wellcome Genome Campus, Hinxton, Cambridgeshire CB10 1SD, UK; European Molecular Biology Laboratory, European Bioinformatics Institute (EMBL-EBI), Wellcome Genome Campus, Hinxton, Cambridgeshire CB10 1SD, UK; European Molecular Biology Laboratory, European Bioinformatics Institute (EMBL-EBI), Wellcome Genome Campus, Hinxton, Cambridgeshire CB10 1SD, UK; European Molecular Biology Laboratory, European Bioinformatics Institute (EMBL-EBI), Wellcome Genome Campus, Hinxton, Cambridgeshire CB10 1SD, UK; European Molecular Biology Laboratory, European Bioinformatics Institute (EMBL-EBI), Wellcome Genome Campus, Hinxton, Cambridgeshire CB10 1SD, UK; European Molecular Biology Laboratory, European Bioinformatics Institute (EMBL-EBI), Wellcome Genome Campus, Hinxton, Cambridgeshire CB10 1SD, UK; European Molecular Biology Laboratory, European Bioinformatics Institute (EMBL-EBI), Wellcome Genome Campus, Hinxton, Cambridgeshire CB10 1SD, UK; European Molecular Biology Laboratory, European Bioinformatics Institute (EMBL-EBI), Wellcome Genome Campus, Hinxton, Cambridgeshire CB10 1SD, UK; European Molecular Biology Laboratory, European Bioinformatics Institute (EMBL-EBI), Wellcome Genome Campus, Hinxton, Cambridgeshire CB10 1SD, UK; European Molecular Biology Laboratory, European Bioinformatics Institute (EMBL-EBI), Wellcome Genome Campus, Hinxton, Cambridgeshire CB10 1SD, UK; European Molecular Biology Laboratory, European Bioinformatics Institute (EMBL-EBI), Wellcome Genome Campus, Hinxton, Cambridgeshire CB10 1SD, UK; European Molecular Biology Laboratory, European Bioinformatics Institute (EMBL-EBI), Wellcome Genome Campus, Hinxton, Cambridgeshire CB10 1SD, UK; European Molecular Biology Laboratory, European Bioinformatics Institute (EMBL-EBI), Wellcome Genome Campus, Hinxton, Cambridgeshire CB10 1SD, UK; European Molecular Biology Laboratory, European Bioinformatics Institute (EMBL-EBI), Wellcome Genome Campus, Hinxton, Cambridgeshire CB10 1SD, UK; European Molecular Biology Laboratory, European Bioinformatics Institute (EMBL-EBI), Wellcome Genome Campus, Hinxton, Cambridgeshire CB10 1SD, UK; European Molecular Biology Laboratory, European Bioinformatics Institute (EMBL-EBI), Wellcome Genome Campus, Hinxton, Cambridgeshire CB10 1SD, UK; European Molecular Biology Laboratory, European Bioinformatics Institute (EMBL-EBI), Wellcome Genome Campus, Hinxton, Cambridgeshire CB10 1SD, UK; European Molecular Biology Laboratory, European Bioinformatics Institute (EMBL-EBI), Wellcome Genome Campus, Hinxton, Cambridgeshire CB10 1SD, UK; European Molecular Biology Laboratory, European Bioinformatics Institute (EMBL-EBI), Wellcome Genome Campus, Hinxton, Cambridgeshire CB10 1SD, UK; European Molecular Biology Laboratory, European Bioinformatics Institute (EMBL-EBI), Wellcome Genome Campus, Hinxton, Cambridgeshire CB10 1SD, UK; European Molecular Biology Laboratory, European Bioinformatics Institute (EMBL-EBI), Wellcome Genome Campus, Hinxton, Cambridgeshire CB10 1SD, UK; European Molecular Biology Laboratory, European Bioinformatics Institute (EMBL-EBI), Wellcome Genome Campus, Hinxton, Cambridgeshire CB10 1SD, UK; European Molecular Biology Laboratory, European Bioinformatics Institute (EMBL-EBI), Wellcome Genome Campus, Hinxton, Cambridgeshire CB10 1SD, UK; European Molecular Biology Laboratory, European Bioinformatics Institute (EMBL-EBI), Wellcome Genome Campus, Hinxton, Cambridgeshire CB10 1SD, UK; European Molecular Biology Laboratory, European Bioinformatics Institute (EMBL-EBI), Wellcome Genome Campus, Hinxton, Cambridgeshire CB10 1SD, UK; European Molecular Biology Laboratory, European Bioinformatics Institute (EMBL-EBI), Wellcome Genome Campus, Hinxton, Cambridgeshire CB10 1SD, UK; European Molecular Biology Laboratory, European Bioinformatics Institute (EMBL-EBI), Wellcome Genome Campus, Hinxton, Cambridgeshire CB10 1SD, UK; European Molecular Biology Laboratory, European Bioinformatics Institute (EMBL-EBI), Wellcome Genome Campus, Hinxton, Cambridgeshire CB10 1SD, UK; European Molecular Biology Laboratory, European Bioinformatics Institute (EMBL-EBI), Wellcome Genome Campus, Hinxton, Cambridgeshire CB10 1SD, UK; European Molecular Biology Laboratory, European Bioinformatics Institute (EMBL-EBI), Wellcome Genome Campus, Hinxton, Cambridgeshire CB10 1SD, UK

## Abstract

The European Nucleotide Archive (ENA, https://www.ebi.ac.uk/ena), maintained at the European Molecular Biology Laboratory's European Bioinformatics Institute (EMBL-EBI) provides freely accessible services, both for deposition of, and access to, open nucleotide sequencing data. Open scientific data are of paramount importance to the scientific community and contribute daily to the acceleration of scientific advance. Outlined here are changes to and updates on the ENA service in 2024, aligning with the broad goals of enhancing interoperability, globalisation of the service and scaling the platform to meet current and future needs.

## Graphical Abstract

**Graphical Abstract:**
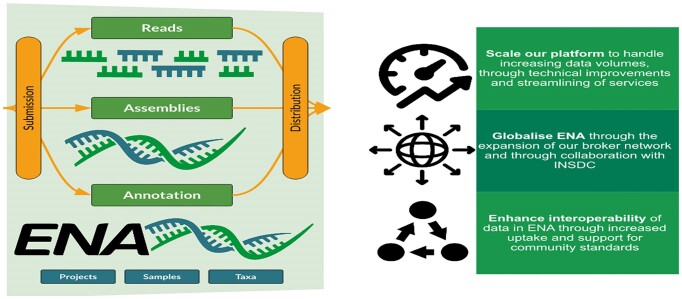

## Introduction

The European Nucleotide Archive(1) (ENA; https://www.ebi.ac.uk/ena) was established in the early 1980s. The ENA offers open data archiving across species, platforms and applications. It is a globally comprehensive record and platform for data coordination with links to other open databases in accordance with FAIR (Findable, Accessible, Interoperable, Reusable) principles (2). The ENA’s services for submission, archival and retrieval are freely available to users across the globe without restriction.

The ENA database is a founding member of the International Nucleotide Sequence Data Collaboration (INSDC) (3), a long-standing global data exchange initiative. The ENA engages with the scientific community, together with our partners in the National Institutes of Health-National Library of Medicine (NIH-NLM) National Center for Biotechnology (NCBI) (4) in the United States and the Research Organization of Information and Systems (ROIS) DNA DataBank of Japan (DDBJ) (5), as trusted custodians of freely available public nucleotide sequence data. The ENA is also recognised as an ELIXIR Core Data Resource (6) and a Global Core Biodata Resource (https://globalbiodata.org/).

Since its foundation, the ENA has developed into a globally critical data platform for the coordination of nucleotide data, and is used in over 90% of the world's countries. Since 2023, significant areas of improvement for the ENA include: enhancing interoperability, scaling the ENA platform and serving a global ENA user base.

### ENA services and challenges

The ENA provides services for the submission, processing, archiving and dissemination of nucleotide sequence data. Data are submitted to the ENA through Webin submission services, and disseminated through the ENA Browser. ENA services are supported by a helpdesk and online user documentation and training materials. The entry points for the key services are listed in Table 1.

**Table 1.** Description of ENA services and their entry points

**Service**	**Description**	**Entry Point**
Data submission	Tools and guidelines to submit or update data with the ENA	https://www.ebi.ac.uk/ena/browser/submit
Data dissemination	Tools and APIs to search, browse, filter and retrieve data from the ENA	https://www.ebi.ac.uk/ena/browser/search
User support' is not valid/accessible	Support form to contact the ENA helpdesk for help or feedback	https://www.ebi.ac.uk/ena/browser/support
Documentation	Guidelines and tutorials on how to use the ENA for data submission and retrieval	https://www.ebi.ac.uk/ena/browser/guides

**Figure 1.**
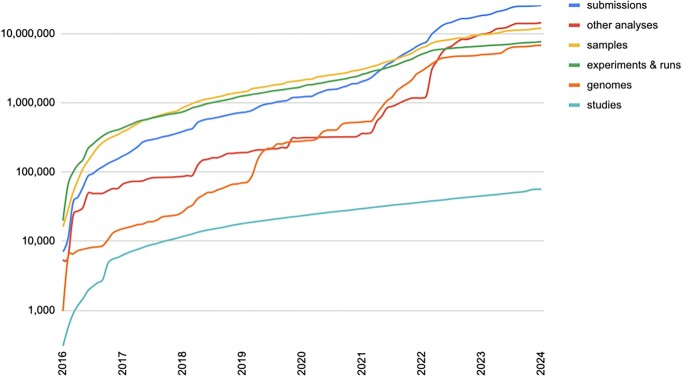
Graph showing growth in ENA submitted public and private data objects over the previous eight years. Suppressed, cancelled or killed objects are not included in the object counts. The ENA sees continual growth in all objects submitted. This graph does not include objects mirrored from INSDC partners.

Continual growth across all ENA submittable objects in 2024 is observed (Figure 1). In the previous 12 months, over 2500 unique submission accounts from 91 countries submitted data to the ENA. In addition to the data traditionally exchanged with our INSDC partners, the ENA received over 12 400 studies, 1.5 million samples, 1.2 million raw read datasets and 500 000 genome assemblies. Accounting for data from across all of the INSDC (i.e. including both ENA submitted data and data mirrored from other INSDC partners), over 32 million raw read datasets are now archived at the ENA and 25 trillion nucleotides worth of sequence information is represented in our assembled and annotated sequence collections. Archived data available to users to download immediately takes up 62.8 petabytes (PB) of storage at EMBL-EBI; this does not account for the additional storage used for backup copies. Based on current trends and technology, this is expected to double in size in just over 3 years.

The ENA Browser is used by 66250 monthly web visitors on average, while programmatic consumers make tens of millions of requests to the our application programming interfaces (APIs) like the ENA Portal API (https://www.ebi.ac.uk/ena/portal/api) and the ENA Browser API (https://www.ebi.ac.uk/ena/browser/api) each month. On average the ENA Portal API had 38.13 million requests from 33 127 unique hosts per month, serving 861 gigabytes (GB) of metadata, while the ENA Browser API served over 45.36 million requests from 42656 unique hosts per month, serving 3.35 terabytes (TB) of metadata and data.

The scale of the ENA in the volume of data submissions and retrieval, the diversity of scientific communities using the service and global reach brings challenges to building and maintaining a service. The work over the past year reflects how these challenges are being addressed. Developments include: work to improve interoperability of the wealth of data by working with standards organisations, delivering service and infrastructure improvements to scale our platform in response to growing data volumes and working towards a global ENA through training, outreach and the brokering network.

### Selected developments in 2024

#### Enhanced interoperability

A sequence database cannot maximise its utility by existing in isolation. To enhance the impact of data archived with the ENA, the service works to improve its approach to interoperable data standards, within the INSDC and externally with data standards organisations.

### Replacement of /country qualifier for sequences

The INSDC introduced mandatory spatiotemporal annotation for new BioSamples (7) in 2023. This had a notable impact on metadata standards. In the four months before the implementation, 70% of BioSamples included country and 52% collection date metadata, whereas in the four months following implementation of the new minimal requirement, 92% of the submitted samples included country and 91% collection date (where the remainder reported missing values). Implementation of this requirement for sequences was undertaken in 2024. Spatiotemporal annotation for sequences required an additional development within the ENA and the INSDC, primarily that the geographical qualifier in sequence flat files ‘/country’ did not comprehensively capture the nature of the data that was expected in the spatiotemporal annotation mandate. For example, the qualifier did not include concepts like ocean names. This year the ‘/country’ qualifier was migrated to a new qualifier called ‘/geo_loc_name’. All qualifiers can be found in the feature table document on the INSDC website (https://www.insdc.org/submitting-standards/feature-table/). This furthers the ENA’s goals of improving the FAIRness of the data in the INSDC and aligning sequence standards with that of samples.

### Major updates to the MIxS sample checklists

The Genomic Standards Consortium (GSC)(8) creates and maintains metadata standards in support of FAIR principles. The prominent standard ‘MIxS’ (Minimal Information About (X) Any Sequence), is used by the INSDC and other databases globally. The standards set by the GSC for MIxS are used by communities to harmonise and improve their data interoperability. As such, there is intrinsic value in the ENA ensuring it synchronises its metadata ecosystem with that promoted by GSC. This year the ENA updated their checklists and field names from MIxS v5 to v6.2. The update includes hundreds of new fields and field definition updates (Table 2). Four new ENA checklists based on MIxS have been created: agriculture, symbiont, food and hydrocarbon.

**Table 2.** Changes to the checklist sample terms

**Count**	**What**
1031	total terms now in ENA
368	new terms
47	aliases added
16	existing definitions updated

### ORCiD data claiming availability in ENA Browser

Over the course of the ENA’s service history, the ways in which user communities interact with and produce data have evolved significantly. Historically, it was standard practice for each submitted sequence or read dataset to be accompanied by a publication. While this remains true for some datasets, there is now a growing trend where data are generated and archived publicly without an associated publication. This shift presents challenges for researchers and contributors in obtaining proper recognition and credit for the nucleotide data they generate and make available to the scientific community.

Open Researcher and Contributor ID (ORCiD) are a global not-for-profit organisation that aims to connect researchers, their contributions and affiliations. To achieve this, ORCiD provides unique and persistent identifiers alongside other related services for users engaging in research, innovation and other activities. ORCiDs have traditionally been associated with scholarly literature publications. Recently the ability to claim datasets shared at EMBL-EBI has also been introduced. A comprehensive list of data resources and the 15 data types that can be claimed is available on the EMBL-EBI website (https://www.ebi.ac.uk/ebisearch/documentation/orcid-claiming#before-starting). Within the ENA, users can claim various data objects, including BioSamples, studies, reads, experiments and sequences.

**Figure 2.**
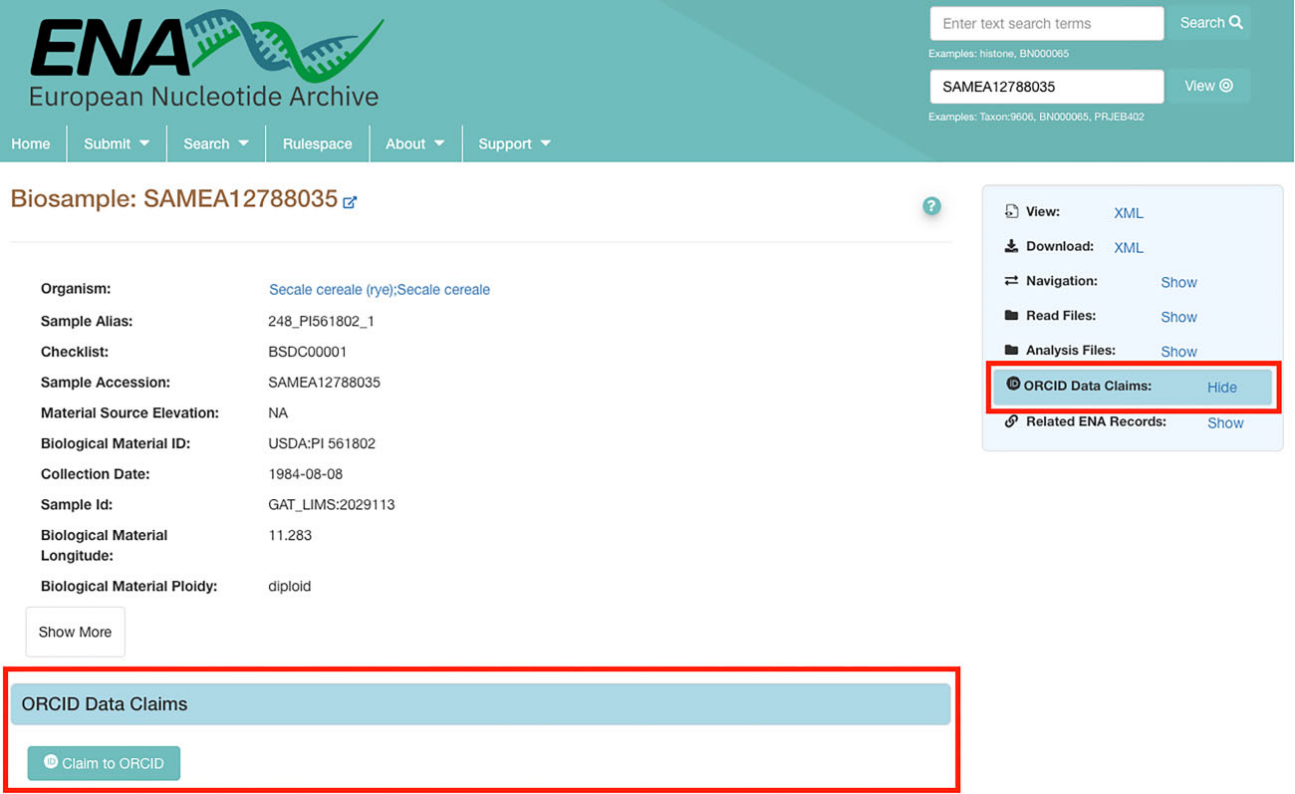
Screenshot of a sample record in the ENA browser: https://www.ebi.ac.uk/ena/browser/view/SAMEA12788035. The right hand side red box presents the new sidebar menu item and the bottom red box presents the ORCID Data Claims section that appears once clicking on the sidebar menu.

To facilitate data claiming, new functionality has been introduced in the ENA browser.The sidebar for individual records now includes an ‘ORCID Data Claims’ button (Figure 2). This enables users to navigate to the ‘ORCID Data Claims’ section of the record page and view existing data claims and/or claim the data object to ORCID through a ‘Claim to ORCID’ button. When users click the ‘Claim to ORCID’ button, they are redirected to the EMBL-EBI page with pre-highlighted entries to claim the dataset. Once claimed, the dataset appears under a user's ‘Works’ section in ORCiD, and their ORCiD appears under the ORCID Data Claims section for the record in the ENA browser.

### Improvements to the tagging system

In 2023, the ENA implemented a tagging system to improve data discoverability, enabling users to explore concise, domain-specific datasets without the need for constructing complex queries. The marine data community serves as a key example of how discoverability requirements have influenced the tagging system's evolution.

‘Marine data’ is a broad term, and identifying a comprehensive dataset within the ENA’s extensive collection would be challenging, requiring in-depth knowledge of metadata and taxonomic expertise. Even with such expertise, constructing complex queries to subset data would be difficult. To address this, enhancements to the tagging system were necessary.

Environmental tags were introduced to provide evidence about the likely environments from which data originated. Currently, two sets of evidence are available, based on quality-controlled, validated sample attributes: taxonomy and geolocation. Taxonomy-based evidence is derived from the World Register of Marine Species (WoRMS) and includes a curated list of ‘metagenome’ taxa from NCBI taxonomy, mapped to three environments: ‘marine,’ ‘freshwater’ and ‘terrestrial.’ Geolocation-based evidence is sourced from geospatial data (shapefiles) that define geographic boundaries for these environments. Future improvements will include a third evidence set, based on free-text, non-validated sample attributes parsed against terms from the Environmental Ontology (ENVO) related to these environments.

**Figure 3.**
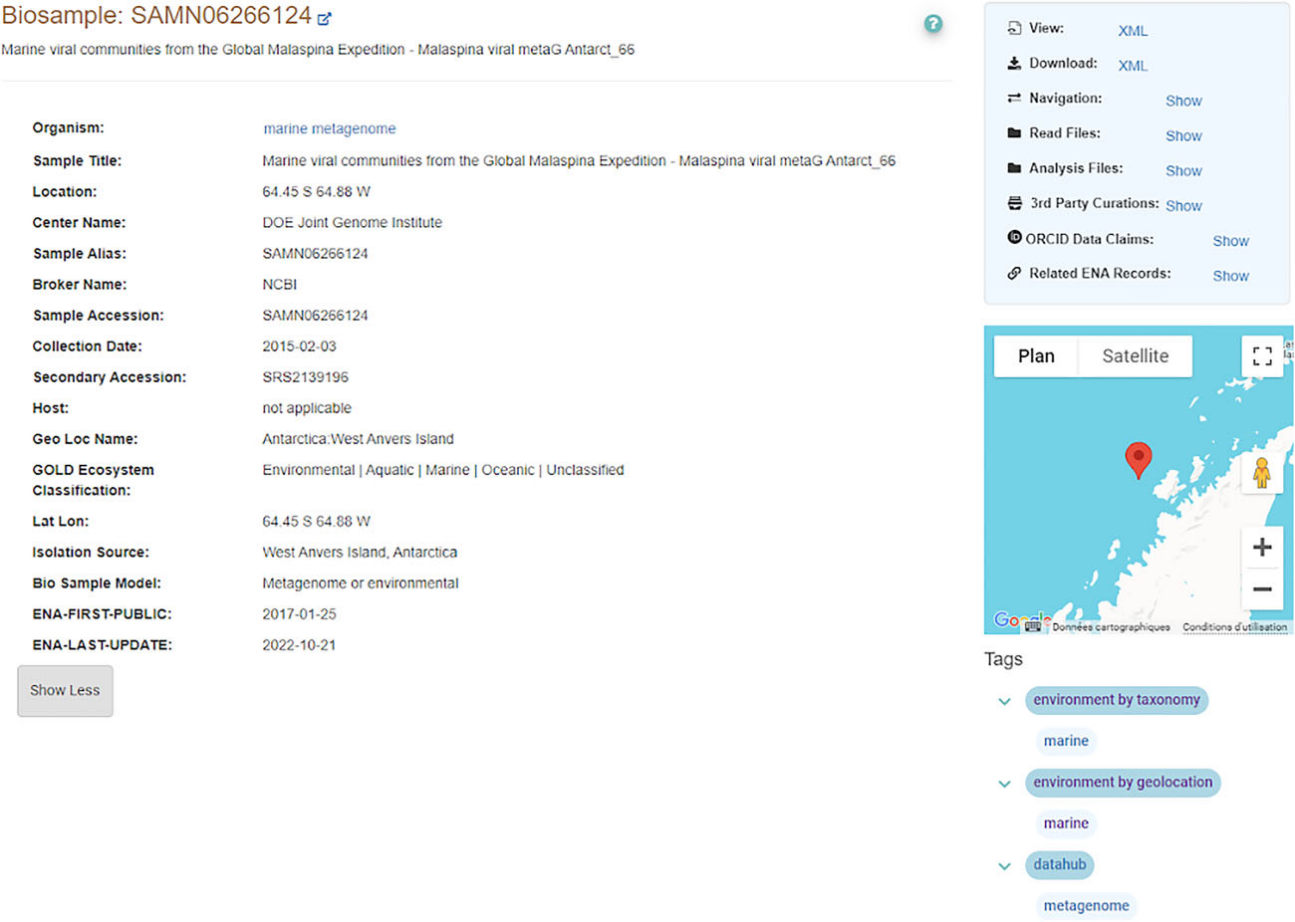
Screenshot of a sample (https://www.ebi.ac.uk/ena/browser/view/SAMN06266124) ENA browser that displays the two sets of environmental tags (below the map) for a sample that was collected in the marine environment.

**Figure 4.**
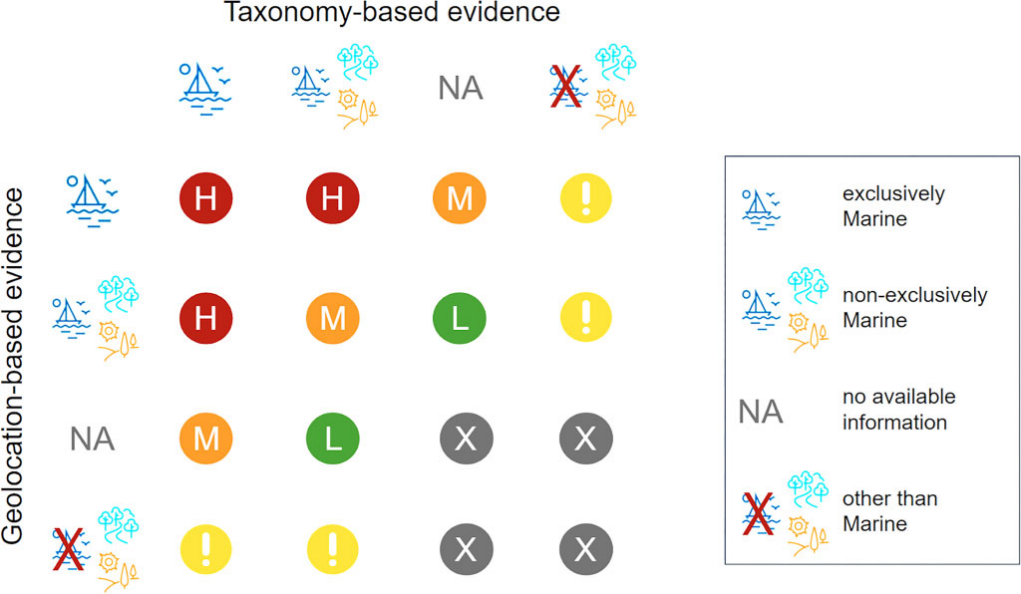
Diagram showing how tags from geolocation- and taxonomy-based evidence are used to assess if an ENA record likely originated from the marine environment with (H)igh, (M)edium or (L)ow confidence levels. (!) Cases where the two sets of evidence disagree can be used in order to investigate possible mistakes in the metadata. (X) Cases where there is no evidence that the record originated from the marine environment.

The two current evidence sets (‘environment by taxonomy’ and ‘environment by geolocation’) and their respective tags—‘marine,’ ‘freshwater’ and ‘terrestrial’—are displayed in the ENA Browser (Figure 3), allowing users to browse records by tag. Users can also construct custom queries in ENA’s advanced search using these environmental tags. Additionally, the ENA provides specific communities with predefined queries (via curl or https), which can, for example, return lists of records originating from the marine environment with high, medium, or low confidence levels. These confidence levels are determined by combining tags from both evidence sets (Figure 4).

### User data journeys

Ensuring data are findable and accessible are central components of making data FAIR at the ENA. Enhancing the user experience in accessing data from the ENA is a key priority. To this end, we have implemented two significant improvements in genome assembly processing and data presentation in the ENA Browser.

Genome assembly processing at the ENA has historically been a complex, lengthy and unreliable process. It could take up to several weeks for a genome assembly to appear in the ENA browser once public. Over the past year, the ENA has made improvements to the genome assembly pipeline. A genome assembly will now display on the ENA browser within 48 hours of going public.

**Figure 5.**
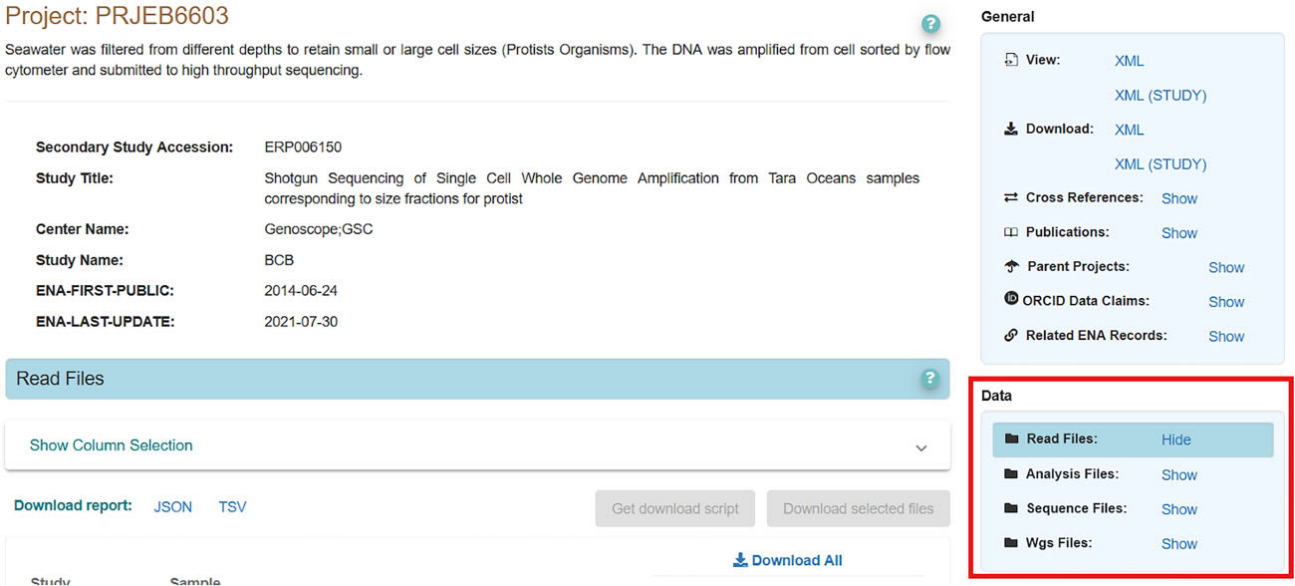
Screenshot of new data centric-view on a project record (https://www.ebi.ac.uk/ena/browser/view/PRJEB6603) from the ENA Browser. The new data focussed box is highlighted in red. Where no public data are available, this box displays a message indicating as such to the user. Traditionally, this would all be contained in the ‘general’ box, with no clear messaging about available public data.

Responding to the growing complexity of data types in ENA, we have moved away from the provision of detailed landing pages for each data type. The ENA browser now separates data files from metadata and related records (Figure 5). This allows users to more quickly find links to associated data files. When no public data are associated with a record, a message will appear to indicate this.

### Service health dashboard

**Figure 6.**
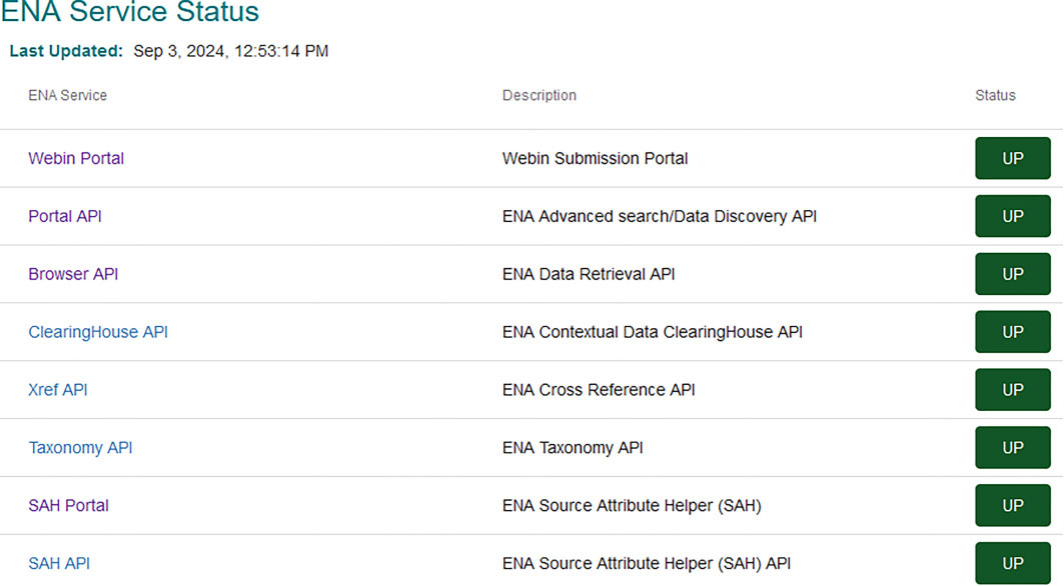
Screenshot of the service health dashboard (https://www.ebi.ac.uk/ena/browser/service-status). This displays to users the name of the service, a description of what the service is responsible for and a live status using ‘UP’ or ‘DOWN’.

To provide users with open and transparent access to our services, we have introduced a service health dashboard (Figure 6).

The service health dashboard allows users to see the status of ENA service entry points, across submission, search and presentation. The dashboard is tied directly to the live analytics of each of the services, which also trigger alerts to ENA team members. This new service gives users the ability to check the status of the relevant service if they encounter an issue, helping users and the ENA team to distinguish between errors caused by transient outages or true issues that need helpdesk support.

### Scaling the platform

#### BioSamples

Submission services at the ENA have fully transferred sample accessioning and archival authority over to the BioSamples service at EMBL-EBI. This has been integrated into all ENA submission endpoints and Webin account authentication has been integrated into BioSamples to maintain account authority and provenance over samples.

Delegating sample authority to BioSamples allows the ENA to rely on a closely connected service at EMBL-EBI, facilitating long-term scalability. Additionally, BioSamples integration allows for better interoperation with other platforms and services that utilise the BioSamples service to create digital representations of their respective physical samples.

### Scaffold submissions

Starting in September 2024, ENA discontinued the acceptance of Assembly Gap Parts (AGP) files describing assembled genomes, with the objective of simplifying how users submit scaffold level assemblies. This change also reduces maintenance by streamlining the submission process for this type of data. Instead of explicit gap definitions in an AGP file, a new declaration system is in place for gaps: strings of Ns are placed in the FASTA file and manifest file's MINGAPLENGTH value is set to an appropriate integer value; each run of Ns which exceeds or matches this length will be recognised as a scaffold.

### Towards decoupled annotation

Development of systems to decouple annotation information from sequence is underway. This will allow the simplification of submission and update transactions, both for users and ENA support operations and will ultimately involve migration away from EMBL Flat File format for capturing submitted sequence data. Instead of taking a single flat file for a sequence, that can either be annotated or unannotated, we will move towards a system of ingesting sequences as FASTA files only, which can be associated with a new sequence annotation object type to be submitted in General Feature Format (GFF3) format. This new model will also allow several different annotation representations associated with a single sequence or assembly, and it will allow users to update annotations independent of sequence versions.

### Supporting a global community

#### Data brokering

Data brokers are institutions or consortia that do not own the data but instead submit it on behalf of, and attribute credit to, the original data producers or owners. Data brokering plays a significant role in how the ENA interacts with its community of data submitters. The ENA continues to expand its data brokering network, which helps scale operations by consolidating points of contact while managing large data volumes. Brokered data submissions also lead to enriched metadata, thereby enhancing the FAIRness of ENA data.

As of now, 58 broker accounts across 18 countries, from as far afield as India and Brazil, have been registered with the ENA. Brokered submissions account for 5% of all public raw sequence reads and 22% of all assembled sequence data currently held by the ENA.

### Communications updates

The ENA continues to enhance its communication with users by strategically engaging with social media to broaden its global visibility. Over the past 12 months, we have focussed on community building through LinkedIn, a platform that has grown in popularity in recent years. LinkedIn allows us to produce both long- and short-form content, reaching a global audience. The ENA currently has approximately 800 followers on the platform.

### Training

Training is a critical aspect of the ENA’s engagement with its user community, serving as a valuable platform for dialogue regarding the utility of our services to the global audience we support. The ENA team has been actively involved in delivering both on-site and off-site training sessions. Notable off-site training events include sessions on data submission and retrieval, conducted as part of the Online Resources and Biological Data Analysis course organised by the University of the Andes in Colombia. Additionally, the ENA provided training for the Intensive Genome Sequencing Course at the University of Konstanz, as well as the Submitting, Finding, and Downloading Raw Sequencing Data course organised by the Swiss Institute of Bioinformatics.

### INSDC global participation initiative

To achieve its mission and vision, the INSDC has established an initiative to allow new membership (https://www.insdc.org/global-participation/). By diversifying participation through new membership, this initiative will advance open science and data sharing and, in turn, drive innovation. Through the inclusion of new members from around the world, the INSDC seeks to contribute to the development of equitable systems for the sharing and dissemination of nucleotide data, while also gaining valuable insights into global trends in genomics.

## Data Availability

No generated data is included here. All data mentioned in the article is publicly available from the European Nucleotide Archive at https://www.ebi.ac.uk/ena/browser/home.

## References

[1] Yuan D. , Ahamed A., Burgin J., Cummins C., Devraj R., Gueye K., Gupta D., Gupta V., Haseeb M., Ihsan M. et al. The European Nucleotide Archive in 2023. Nucleic Acids Res. 2023; 52:D92–D97. 10.1093/nar/gkad1067 PMC10767888 37956313

[2] Wilkinson M.D. , Dumontier M., Aalbersberg I.J.J., Appleton G., Axton M., Baak A., Blomberg N., Boiten J.-W., da Silva Santos L.B., Bourne P.E. et al. The FAIR Guiding Principles for scientific data management and stewardship. Sci. Data. 2016; 3:160018. 26978244 10.1038/sdata.2016.18 PMC4792175

[3] Arita M. , Karsch-Mizrachi I., Cochrane G. The international nucleotide sequence database collaboration. Nucleic Acids Res. 2021; 49:D121–D124. 33166387 10.1093/nar/gkaa967 PMC7778961

[4] Sayers E.W. , Cavanaugh M., Clark K., Pruitt K.D., Schoch C.L., Sherry S.T., Karsch-Mizrachi I. GenBank. Nucleic Acids Res. 2021; 49:D92–D96. 33196830 10.1093/nar/gkaa1023 PMC7778897

[5] Ogasawara O. , Kodama Y., Mashima J., Kosuge T., Fujisawa T. DDBJ Database updates and computational infrastructure enhancement. Nucleic Acids Res. 2020; 48:D45–D50. 31724722 10.1093/nar/gkz982 PMC7145692

[6] Drysdale R. , Cook C.E., Petryszak R., Baillie-Gerritsen V., Barlow M., Gasteiger E., Gruhl F., Haas J., Lanfear J., Lopez R. et al. The ELIXIR Core Data Resources: fundamental infrastructure for the life sciences. Bioinformatics. 2020; 36:2636–2642. 31950984 10.1093/bioinformatics/btz959 PMC7446027

[7] Courtot M. , Cherubin L., Faulconbridge A., Vaughan D., Green M., Richardson D., Harrison P., Whetzel P.L., Parkinson H., Burdett T. BioSamples database: an updated sample metadata hub. Nucleic Acids Res. 2019; 47:D1172–D1178. 30407529 10.1093/nar/gky1061 PMC6323949

[8] Field D. , Amaral-Zettler L., Cochrane G., Cole J.R., Dawyndt P., Garrity G.M., Gilbert J., Glöckner F.O., Hirschman L., Karsch-Mizrachi I. et al. The Genomic Standards Consortium. PLoS Biol. 2011; 9:e1001088. 21713030 10.1371/journal.pbio.1001088 PMC3119656

